# Noninvasive Measurement of Pulmonary Function in Experimental Mouse Models of Airway Disease

**DOI:** 10.1007/s00408-021-00443-9

**Published:** 2021-05-19

**Authors:** Thomas Glaab, Armin Braun

**Affiliations:** 1 Department of Internal Medicine III Hematology, Oncology, Pneumology, University Medical Center Mainz, Mainz, Germany; 2 Division Preclinical Pharmacology and Toxicology, Member of the German Center for Lung Research (DZL), Biomedical Research in Endstage and Obstructive Lung Disease (BREATH) Research Network, Member of Fraunhofer International Consortium for Anti-Infective Research (iCAIR), Fraunhofer Institute for Toxicology and Experimental Medicine (ITEM), Nikolai-Fuchs-Str. 1, 30625 Hannover, Germany; 3 Institute of Immunology, Hannover Medical School, Hannover, Germany

**Keywords:** Pulmonary function testing, Allergic asthma, Airway hyperresponsiveness, Animal models, SARS-CoV-2

## Abstract

Mouse models have become an indispensable tool in translational research of human airway disease and have provided much of our understanding of the pathogenesis of airway disease such as asthma. In these models the ability to assess pulmonary function and particularly airway responsiveness is critically important. Existing methods for testing pulmonary function in mice in vivo include noninvasive and invasive technologies. Noninvasive head-out body plethysmography is a well-established and widely accepted technique which has been proven as a reliable method to measure lung function on repeated occasions in intact, conscious mice. We have performed several validation studies in allergic mice to compare the parameter midexpiratory flow (EF_50_) as a noninvasive marker of airflow limitation with invasively measured gold standard parameters of lung mechanics. The results of these studies showed a good agreement of EF_50_ with the invasive assessment of lung resistance and dynamic compliance with a somewhat lower sensitivity of EF_50_. The measurement of EF_50_ together with basic respiratory parameters is particularly appropriate for simple and repeatable screening of pulmonary function in large numbers of mice or if noninvasive measurement without use of anesthesia is required. Beyond known applications, head-out body plethysmography also provides a much-needed high-throughput screening tool to gain insights into the impact and kinetics of respiratory infections such as SARS-COV-2 on lung physiology in laboratory mice.

## Introduction

The ability to measure pulmonary function in individual mice on repeated occasions is of great interest because of the prominent role played by these animals into the causes and mechanisms of respiratory and allergic diseases such as asthma. This is largely due to relatively low maintenance costs, a well characterized genome and immune system, high breeding efficiency, the large availability of inbred and transgenic strains, and the technologies for genetic manipulation not yet available in other animals. Mouse models of experimental asthma have contributed substantially to our understanding of pathomechanisms underlying allergic airway inflammation and airway hyperresponsiveness (AHR) [1–3]. However, murine models of airway disease also have limitations that need to be taken into account when extrapolating findings from the animal model to the human disease. The potential advantages of allergic mouse models as well as relevant differences between mouse and human immunology, anatomy and physiology have been reviewed in the literature[1, 4].

## Lung Function Measurements in Mice: Strengths and Limitations

AHR defined as the predisposition of the airways to react excessively to bronchoconstrictor agents is an important component of the asthma phenotype. Pulmonary function tests are important to assess lung function and airway responsiveness (AR) in intact organisms, but the development of these tests has been a great challenge due to the small size of murine airways. In recent years, considerable progress has been made in developing valid and suitable pulmonary function measurements [5–8]. Current methods that assess murine pulmonary function can be categorized broadly into invasive and noninvasive techniques.

It is important to recognize that each approach represents a compromise between accuracy, noninvasiveness, and convenience. As a result, a correlation exists between the invasiveness of a measurement technique and its precision [9]. The less invasive a measurement, the less likely it is to produce consistent, reproducible and meaningful data. Invasive monitoring of lung function using parameters such as pulmonary resistance (R_L_) or dynamic compliance (C_dyn_) is the classical method for accurate and specific determination of pulmonary mechanics. The low-frequency forced oscillation technique (LFOT) is currently considered to be the most precise technology to determine both central airway resistance and peripheral tissue compliance. [5, 10, 11]. The primary drawback of the invasive techniques involving tracheostomy is the inability to make repeated measurements. This limitation has been overcome by the use of an orotracheal intubation technique, allowing for repetitive monitoring of airway mechanics in the same animals [12, 13]. However, this modification still requires anesthesia and is technically challenging. To circumvent the significant challenges associated with invasive measures, more convenient but less specific noninvasive plethysmographic methods have been described [14–16]. Concerns with existing noninvasive methods in spontaneously breathing mice include the contribution of upper airway resistance (changes of glottal aperture, nasal passages) and the uncertainty about the exact magnitude and site of bronchoconstriction [8].

Results of our previous studies suggest that midexpiratory tidal flow (EF_50_), as measured by head-out body plethysmography, can be used as a noninvasive measure of bronchoconstriction in mice and rats [16–18]. The assessment of EF_50_ in conscious animals has a number of advantages. First, the measurements are technically easy to perform. Second, because this method does not require anesthesia or tracheal interventions, the same animal can be studied on multiple occasions over an extended period of time.

This review reflects our own practical experience with invasive and noninvasive lung function methods in mouse models of allergic inflammation. In this context, we have provided first experimental evidence that the noninvasive determination of EF_50_ can be used as a valid physiological measure of nonspecific and allergen-specific airway hyperresponsiveness in conscious mice. In addition, this article will also focus on the feasibility, further applications and limitations of noninvasive pulmonary function testing using head-out body plethysmography.

## Noninvasive Head-Out Body Plethysmography

**Fig. 1:**
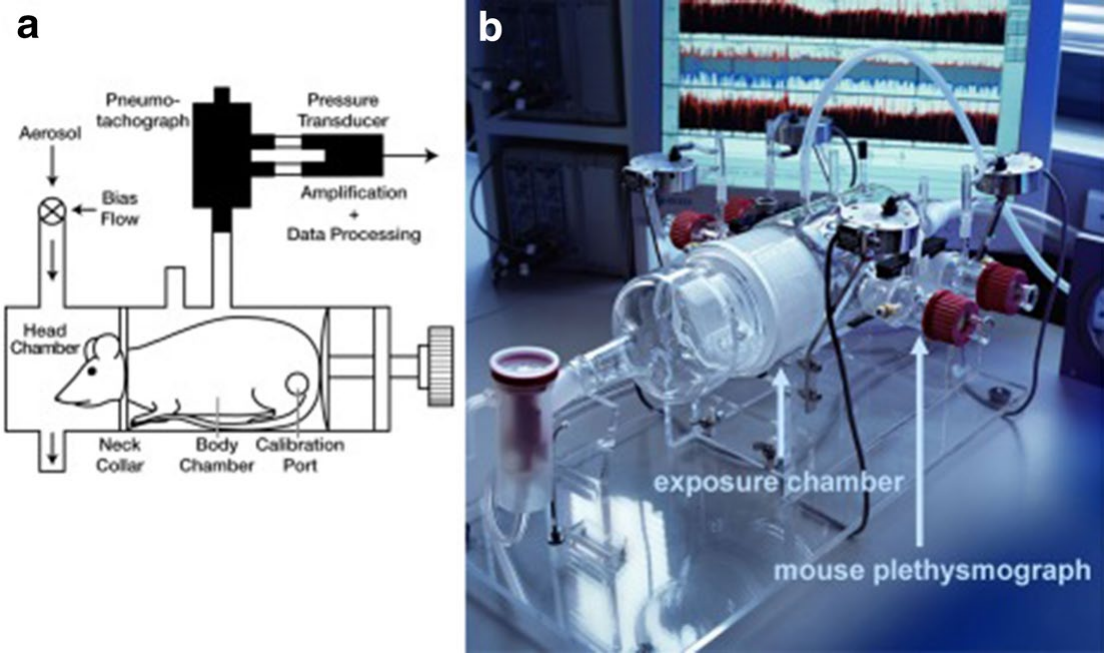
Head-out body plethysmography. **a** Illustration of a head-out body plethysmography system. Figure taken from [17] with permission, modified. The single chambers are attached to a head exposure chamber. Airflow is recorded via a pneumotachograph connected to a pressure transducer. **b** Image of a head-out body plethysmography system for four mice. Figure taken from [20] with permission, modified

With head-out body plethysmography, animals acclimatized to the chambers are gently placed in the glass body plethysmographs while the head of each animal protrudes through a neck collar into a ventilated head exposure chamber (Fig. 1) [8, 16–20]. Working under conditions where the animals are comfortable, well adapted to the chamber (prior acclimatization training, e.g., 5 days in increasing time periods), start of measurement when mice and respiratory parameters settled down to a stable level and a quiet working environment are beneficial in terms of stress-induced catecholamine release, test variability and quality. Basically, head-out body plethysmography simply measures the air being displaced by the animal`s expanding and contracting thorax and obviates efforts to compensate for the adiabatic conditions that occur through temperature and humidity changes by inspired and expired air of mice placed in a whole-body plethysmography chamber. A continuous bias flow through the head chamber further allows continuous acute and long-term measurements of pulmonary function with no need to replace the air inside the plethysmographs. Aerosols can be delivered directly through the head exposure chamber. Tidal flow is measured by a pneumotachograph connected to a differential pressure transducer that are attached to each body chamber. From these amplified and digitized flow signals several respiratory parameters, including EF_50_ (tidal midexpiratory airflow, indicates airflow limitation), TI (time of inspiration), TE (time of expiration), f (breathing frequency), VT (tidal volume), MV (minute volume), TB (time of braking, indicates sensory irritation), TP (time of pause before inspiration, indicates pulmonary irritation), PIF (peak inspiratory flow), PEF (peak expiratory flow) are derived from software analysis (e.g., Scireq, Buxco, Hugo Sachs Elektronik).

**Fig. 2:**
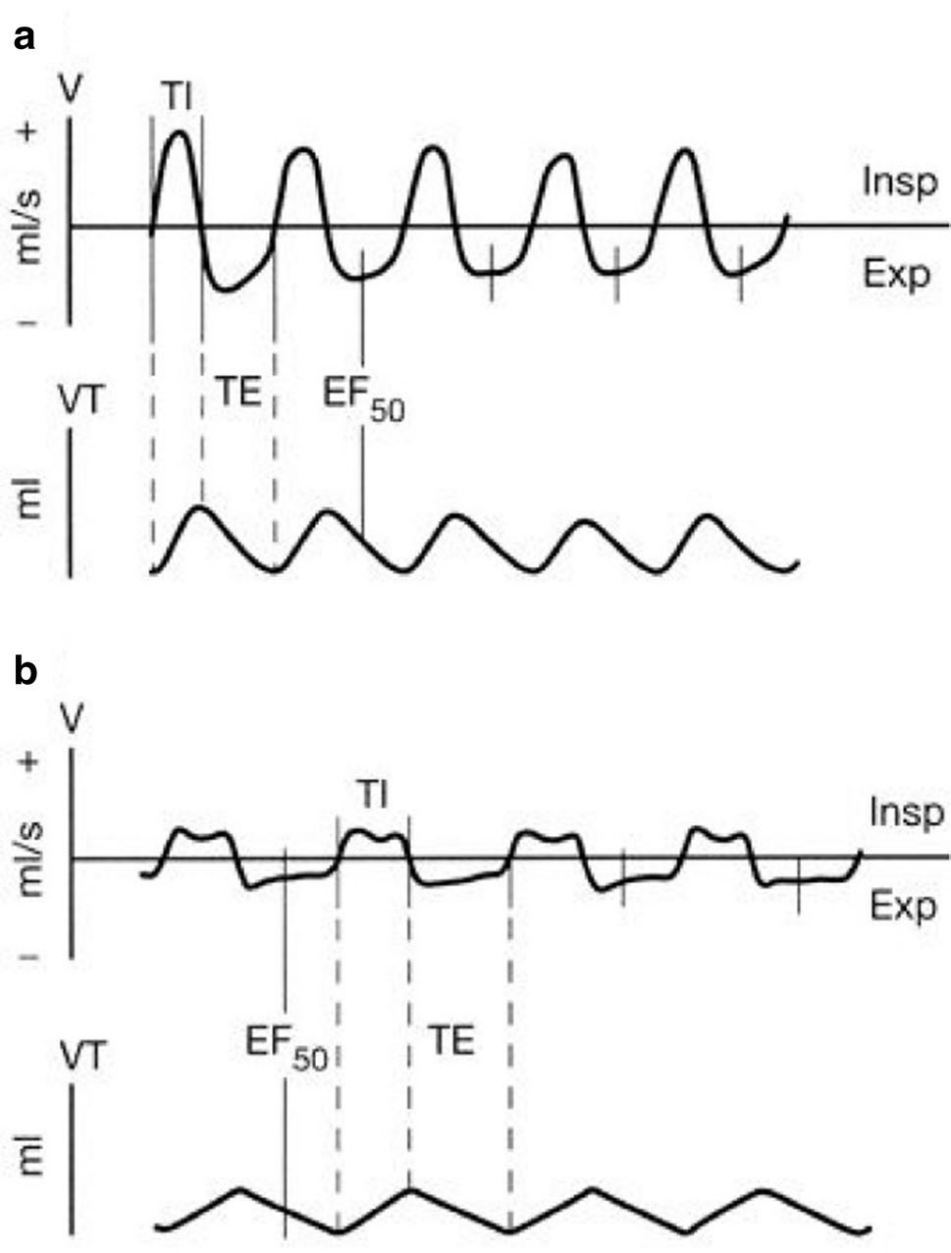
Characteristic breathing patterns of spontaneously breathing, conscious BALB/c mice. **a** Normal breathing pattern. **b** Characteristic pattern of airway obstruction in response to inhaled MCh, showing the decrease in EF_50_. **a** and **b**, top tracings: airflow signal. **a** and **b**, bottom tracings: VT signal. A horizontal line at zero flow separates inspiratory (Insp; upward; +) from expiratory (Exp; downward; −) airflow. V, tidal flow. VT, tidal volume. TI, time of inspiration. TE, time of expiration. Figure taken from [17] with permission

Yves Alarie and coworkers were the first to demonstrate that the noninvasive measurement of midexpiratory flow (EF_50_) as measured by head-out body plethysmography is appropriate to assess airflow limitation in conscious mice [19]. Using head-out body plethysmography, airway narrowing causes characteristic alterations in the breathing pattern, which are best reflected by a reduction in tidal midexpiratory flow (EF_50_, [ml/s]) (Fig. 2). Changes in EF_50_ are typically associated with decreases in tidal volume (VT), breathing frequency (f) and an increase in time of expiration (TE). This noninvasive approach is particularly useful to measure several animals at a time, to monitor pulmonary function repeatedly, and to capture the kinetics of a response over time. Furthermore, the method is straightforward and can be learned in a relatively short time.

In general, the majority of experimental asthma models usually determine the following outcomes: immunological (IgE, IgG, cytokines), histopathological (airway inflammation, remodeling) and functional (pulmonary function, cholinergic and allergen-specific airway hyperresponsiveness). Several validation studies were performed to compare the utility of EF_50_ with invasively recorded gold standard parameters in established mouse models of allergic airway inflammation.

## Validation of EF_50_ vs. Invasive Measurements of Pulmonary Mechanics in Allergic Mice

To evaluate the sensitivity of EF_50_ as measured by head-out body plethysmography and to detect changes in pulmonary function, we measured the bronchoconstrictive response to aerosolized methacholine (MCh) and differentiated between normal levels of airway reactivity in control animals and airway hyperresponsiveness in Ovalbumin (Ova)-sensitized and -challenged BALB/c mice [17].

The EF_50_ response to aerosolized MCh (24 h after allergen challenge) in OVA-sensitized and challenged animals was both shifted to the left and amplified compared with that in control animals. The MCh-related decrease in EF_50_ is presumably mediated by specific muscarinic M3-receptor activation on airway smooth muscles as shown by the rapid onset and resolution of the response to aerosolized MCh.

Decreases in EF_50_ values in allergen-sensitized and -challenged mice were associated with the production of allergen-specific IgE and IgG1 and the development of eosinophil infiltration in the lungs. In addition, increased numbers of eosinophils and lymphocytes as well as elevated titers of the Th2 cytokines IL-4 and IL-5 in broncho-alveolar (BAL) fluid were detected showing a Th2-skewed adaptive immune response. Importantly, EF_50_ closely reflected the enhanced airway response to aerosolized MCh observed with simultaneously measured pulmonary conductance (G_L_ = 1/R_L_) and dynamic compliance (C_dyn_) in allergen-sensitized and -challenged BALB/c mice. We further demonstrated that the decline in EF_50_ to MCh challenge was partly inhibited by pretreatment with an inhaled beta-2-agonist and that associated changes in f and VT did not directly influence EF_50_ values. A limitation of the current validation investigation particularly has included pleural catheterization to measure invasive pulmonary mechanics (G_L_ and C_dyn_) and the contribution of upper airway resistance. This methodological shortcoming introduced variability including uncertainty of the site of obstruction into the results which made them difficult to compare with other invasive techniques [21].

To overcome this limitation we developed and validated a plethysmograph system for invasive lung function measurement combined with an integrated computer-controlled aerosol delivery system [12].This novel in vivo method, for the first time, combined direct and repetitive recordings of standard pulmonary mechanics in anesthetized orotracheally intubated spontaneously breathing mice. This technology represented a substantial advance in accuracy over the previous validation experiments in that G_L_, C_dyn_ and EF_50_ were measured simultaneously in intact mice including local aerosol challenges via an orotracheal tube. The primary objective of this follow-up study in a mouse model of fungal asthma (Aspergillus fumigatus) was to compare the capability of noninvasive EF_50_ measurements to reflect the allergen-specific and cholinergic AR as observed with invasive determination of pulmonary mechanics [16]. Groups were separated into invasively and noninvasively measured allergic and control mice.

**Fig. 3:**
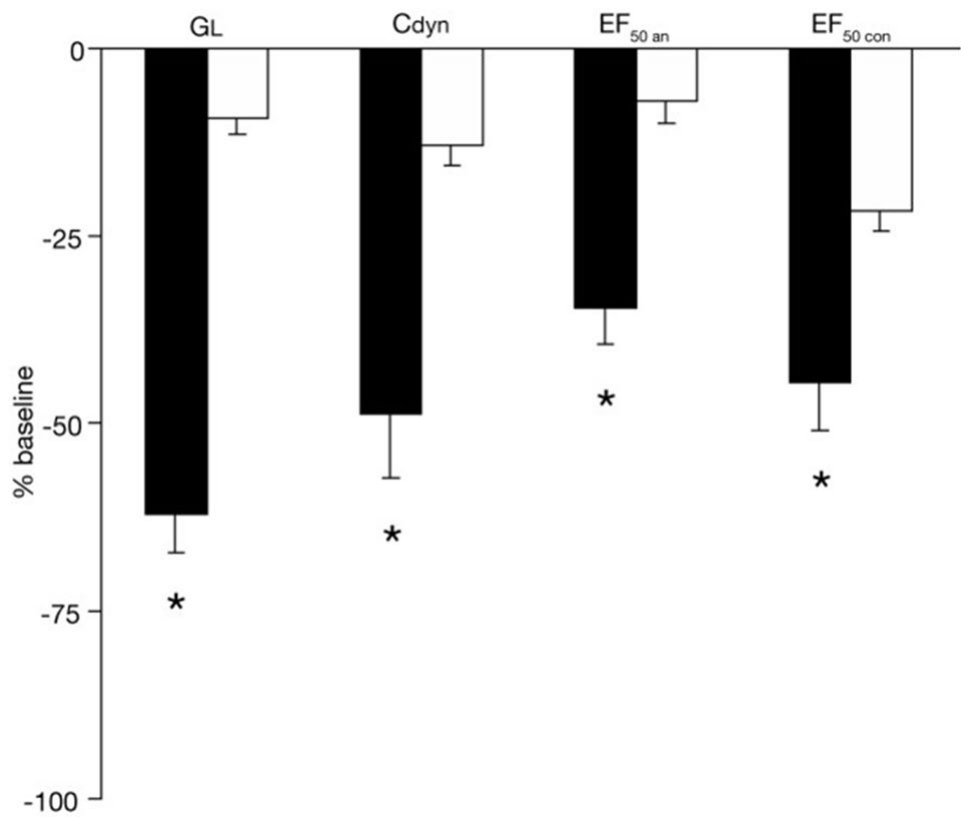
Early airway responsiveness (EAR). Noninvasive vs. invasive assessment of airway hyperresponsiveness to inhaled allergen. Groups were separated into invasively and noninvasively measured allergic and control animals. In contrast to controls, the allergic mice demonstrated significant decreases in simultaneously measured G_L_, C_dyn_ and EF_50_an (an: anesthetized) in response to the specific allergen challenge with inhaled A. fumigatus. The noninvasive recording of EF_50_con (con: conscious) also showed significant reductions in EF_50_ to inhaled A. fumigatus challenge in allergic mice vs. controls. EAR was expressed as % change from baseline values, which were taken as 0%. Values are means ± SE, *n* = 8 per group, *p < 0.01 vs. control. Figure taken from [16] with permission

**Fig. 4:**
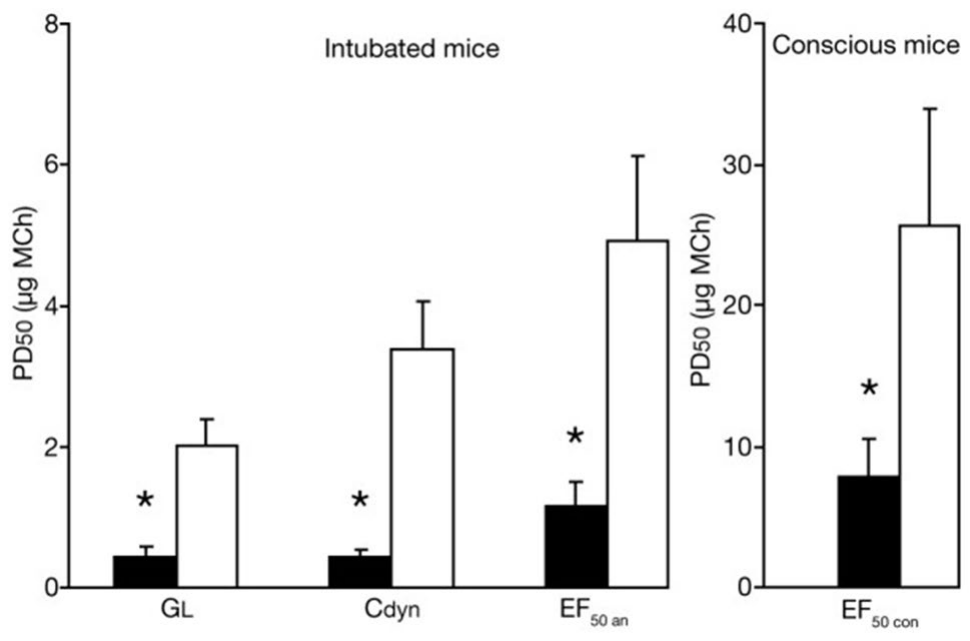
Assessment of cholinergic airway responsiveness 48 h after EAR, expressed as PD50 values (which is the dose of methacholine (MCh) required to reduce either G_L_, C_dyn_ or EF_50_ to 50% of their respective baseline values) of invasively measured G_L_, C_dyn_ and EF_50_ (left-hand side) as well as of noninvasively measured EF_50_ (right-hand side). Allergic mice (black columns) showed significantly lower PD50 values vs. control animals (white columns). Baseline values did not significantly alter from initial baseline values 48 h before. Values are means ± SE, *n* = 8 per group, *p < 0.05 vs. control. Figure taken from [16] with permission

With both methods, allergic mice sensitized and boosted with A. fumigatus revealed allergen-specific early airway responsiveness (EAR) in response to aerosolized allergen, whereas sham-exposed controls were unresponsive (Fig. 3). 48 h later, dose–response studies to inhaled MCh in the same animals demonstrated cholinergic AHR in allergic mice (Fig. 4) vs. controls. Cholinergic AHR was associated with an enhanced influx of neutrophils and eosinophils in BAL fluid.

These outcomes suggest that EF_50_ can discriminate the degree of AR and reflects the changes in invasively recorded G_L_ and C_dyn_ during bronchoconstriction. Moreover, the relation of the cholinergic EF_50_ response between allergic and control animals was similar for invasive and noninvasive measurements. Compared with G_L_ and C_dyn_ recordings, invasive and noninvasive recordings of EF_50_ were less sensitive in detecting the maximum degree of bronchoconstriction during challenges with inhaled allergen and MCh [16–18].

The results of these studies showed a good correlation of the EF_50_ method with the classical parameters of lung mechanics, and are thus particularly suitable for simple and repeatable screening of pulmonary function in large animal numbers on multiple occasions. Since EF_50_ may underestimate the degree of bronchoconstriction it is still unclear how much this limits its use in detecting less pronounced changes of AR. Accordingly, when such circumstances are present, measurements should be validated against invasive standard measures of lung mechanics.

## Comparison of EF_50_ with Penh

In barometric whole-body plethysmography unrestrained mice are placed in a whole-body plethysmograph and the pressure changes that occur in the chamber during the breathing cycle are analyzed. From the box pressure signal during inspiration and expiration, and the timing comparison of early and late expiration, a dimensionless parameter called "enhanced pause" (Penh) has been calculated [15]. Current consensus is that Penh values reflect breathing patterns rather than lung mechanics, and they do not correlate well with conventional parameters of pulmonary mechanics [22–27]. Furthermore, various experimental conditions completely unrelated to lung mechanics such as humidification and warming of inspired gas, hyperoxia, and the timing of ventilation, can also affect Penh [23, 25]. These more careful investigations have thus led to a justifiable skepticism for using Penh as a reliable measure of airway mechanics [26, 27].

Compared with Penh, EF_50_ differs substantially in several important ways: EF_50_ declines with bronchoconstriction and in line with invasive parameters of lung mechanics is associated with a decrease in tidal volume during bronchoconstriction [16, 17]. In addition, EF_50_ is a physiological variable with a physical meaning (ml/s), enables direct comparison from one animal to another and is closely related to airway resistance. By contrast, Penh is not a valid measure of airway resistance and its usefulness was further weakened by a study showing that changes in Penh were no better than simply measuring time of expiration (TE) in various strains of mice [22]. That EF_50_ measurements are independent of f, TE, and VT was determined in studies with various airborne stimuli (CBC and n-propranolol) in conscious animals in which EF_50_ values were unaffected by irritant induced changes in f, VT, and TE, suggesting that EF_50_ does not correlate simply with changes in breathing patterns [19]. These data support the view that EF_50_ more reliably reflects airway resistance than Penh, which is largely a function of respiratory timing.

## Further Applications of Noninvasive Head-Out Body Plethysmography

Beyond known applications in asthma research, integrated use of head-out body plethysmography might contribute to a better understanding of other respiratory disorders including, e.g., experimental models of COPD/emphysema, lung fibrosis, tests on airborne irritant/pollutant effects [8, 19, 28] and respiratory safety pharmacology studies (Phase I) [20]. It is notable that quite a number of standard respiratory variables (f, VT, TI, TE, MV etc.) can be already derived from simple breathing patterns of conscious, spontaneously breathing mice as mentioned before.

In addition, head-out body plethysmography can provide noninvasive and repeatable long-term monitoring of disease responses in the same animals throughout the course of bacterial and viral infections which may propel new ways toward exploring pathogenesis and new drug therapies. As an example, head-out body plethysmography has been reported to be very useful for monitoring infection with Pseudomonas aeruginosa in mice showing a decrease in VT and EF_50_ [29]. Even more interestingly in the light of the current SARS-CoV-2 pandemic, lung function measurement including EF_50_ discriminated both virus and dose-specific responses and identified long-term respiratory changes in experimental mouse models of coronavirus and Influenza A virus infection [30]. Another recent study demonstrated a significant loss of pulmonary function including EF_50_ in a mouse model of SARS-CoV-2 infection [31]. This approach may help to understand the functional outcomes of viral infections such as SARS-CoV-2 on pulmonary function, indicate disease severity prior to traditional pathogenic markers like weight loss and lethality, develop novel animal models of emerging viral respiratory pathogens, and may also provide insights into the treatment, resolution, and long-term physiological impacts of infection. Together, the results underscore the utility of recording respiratory function via body plethysmography also within the context of acute respiratory infections in vivo.

## Conclusion

Although still underutilized, pulmonary function testing has gained popularity due the recognition of its clear value in the functional assessment of experimental models of various acute and chronic respiratory disorders, including allergic asthma. However, at present, there is no gold standard for measuring pulmonary function in mice, because none of the available invasive and noninvasive methods is optimal in all regards. Some investigations (e.g., with potential impact on therapeutic studies in humans) require the most sensitive and reliable determination of lung mechanics in ventilated, anesthetized animals. The noninvasive assessment of respiratory standard metrics is a complimentary approach for screening pulmonary function and the kinetics of respiratory changes in large numbers of conscious mice. We and others have shown that head-out body plethysmography is a valid, reliable and cost-effective method, that can fulfill an important need in many respiratory research applications. The ability to assess and analyze standard respiratory function parameters concurrently with an accepted index of airflow limitation on multiple occasions in the same animal makes this method particularly useful for acute and longitudinal studies without the use of anesthesia. Beyond known applications, head-out body plethysmography will also provide a much-needed high-throughput screening tool for noninvasively evaluating the impact of SARS-Cov-2 infections on pulmonary function and antiviral drug performance in wild-type and genetically altered mice.
